# Supplementary material for: A Novel Method for the Evaluation of Bone Marrow Samples from Patients with Pediatric B-Cell Acute Lymphoblastic Leukemia—Multidimensional Flow Cytometry
Source: Cancers (Basel). 2021 Oct 9;13(20):5044. doi: 10.3390/cancers13205044 (PMC8533788; doi:10.3390/cancers13205044)
Supplement: Supplementary file 1 [file cancers-13-05044-s001.zip › cancers-1379556-supplementary.pdf]

Supplementary Figure S1. Changes in antibody combination during the examined period March 2013 and October 2020

During the examined period, there were some changes in the applied antibody combinations according to the new gudelines. Abbreviations: FITC: fluorescein isothiocyanate, PE: phycoerythrin, PerCP-Cy5.5: peridinin chlorophyll protein cyanine 5.5, PE-Cy7: phycoerythrin cyanine 7, APC: allophycocyanin, APC-H7 allophycocyanin H7, PB: pacific blue, PO: pacific orange

March 2013-October 2017

	FITC	PE	PerCP-Cy5.5/PC5.5	PE-Cy7	APC	APC-H7	PB	PO
Tube 1	syto16	CD66c	CD34	CD19	CD10	CD38	CD20	CD45
Tube 2	CD58	CD123	CD33	CD19	CD10	CD81	syto40	CD45

October 2017-June 2020

	FITC	PE	PerCP-Cy5.5/PC5.5	PE-Cy7	APC	APC-H7	PB	PO
Tube 1	syto16	**CD66c+CD123**	CD34	CD19	CD10	CD38	CD20	CD45
Tube 2	CD58	**CD73+CD304**	CD33	CD19	CD10	CD81	syto40	CD45

June 2020-October 2020

	FITC	PE	PerCP-Cy5.5/PC5.5	PE-Cy7	APC	APC-H7	PB	PO
Tube 1	syto16	**CD66c+CD123**	CD34	CD19	CD10	CD38	CD20	CD45
Tube 2	CD58	**CD73+CD304**	CD33	CD19	CD10	**CD22**	syto40	CD45
